# 6-Chloro-2-cyclo­propyl-4-(trifluoro­meth­yl)quinoline

**DOI:** 10.1107/S1600536811003746

**Published:** 2011-02-05

**Authors:** H. C. Devarajegowda, H. K. Arunkashi, Suresh Babu Vepuri, N. Chidananda, V. D. Jagadeesh Prasad

**Affiliations:** a Department of Physics, Yuvaraja’s College (Constituent College), University of Mysore, Mysore 570 005, Karnataka, India; b Department of Pharmaceutical Chemistry, GITAM Institute of Pharmacy, GITAM University, Visakhapatnam 530 045, Andhrapradesh, India; c Department of Chemistry, Mangalore University, Mangalagangotri 574 199, Karnataka, India

## Abstract

In the title compound, C_13_H_9_ClF_3_N, the quinoline ring system makes a dihedral angle of 88.8 (2)° with the cyclo­propyl ring.

## Related literature

For general background to quinolines see: Kayser & Novak (1987 ▶); Rudin *et al.* (1984 ▶); Mao *et al.* (2009 ▶); Bermudez *et al.* (2004 ▶); Jayaprakash *et al.* (2006 ▶); Andries *et al.* (2005 ▶). For related structures, see: Skörska *et al.* (2005 ▶); Devarajegowda *et al.* (2010 ▶); Li *et al.* (2005 ▶).
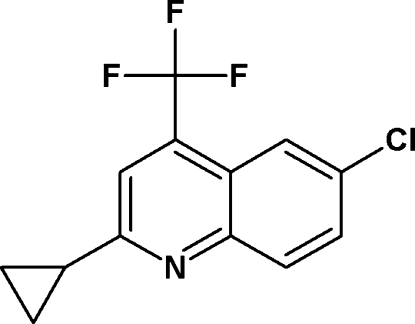


## Experimental

### 

#### Crystal data

C_13_H_9_ClF_3_N
*M* *_r_* = 271.66
Monoclinic, 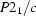

*a* = 13.8482 (19) Å
*b* = 5.0534 (8) Å
*c* = 18.048 (3) Å
β = 107.503 (17)°
*V* = 1204.5 (3) Å^3^
*Z* = 4
Mo *K*α radiation
μ = 0.33 mm^−1^
*T* = 293 K
0.22 × 0.15 × 0.12 mm

#### Data collection

Oxford Diffraction Xcalibur diffractometer
Absorption correction: multi-scan (*CrysAlis PRO RED*; Oxford Diffraction, 2010 ▶) *T*_min_ = 0.942, *T*_max_ = 0.961
11631 measured reflections
2105 independent reflections
946 reflections with *I* > 2σ(*I*)
*R*_int_ = 0.092

#### Refinement

*R*[*F* ^2^ > 2σ(*F* ^2^)] = 0.041
*wR*(*F* ^2^) = 0.081
*S* = 0.78
2105 reflections
164 parameters
H-atom parameters constrained
Δρ_max_ = 0.13 e Å^−3^
Δρ_min_ = −0.17 e Å^−3^

### 

Data collection: *CrysAlis PRO CCD* (Oxford Diffraction, 2010 ▶); cell refinement: *CrysAlis PRO CCD*; data reduction: *CrysAlis PRO RED* (Oxford Diffraction, 2010 ▶); program(s) used to solve structure: *SHELXS97* (Sheldrick, 2008 ▶); program(s) used to refine structure: *SHELXL97* (Sheldrick, 2008 ▶); molecular graphics: *ORTEP-3* (Farrugia, 1997 ▶) and *CAMERON* (Watkin *et al.*, 1993) ▶; software used to prepare material for publication: *WinGX* (Farrugia, 1999 ▶).

## Supplementary Material

Crystal structure: contains datablocks I, global. DOI: 10.1107/S1600536811003746/wn2420sup1.cif

Structure factors: contains datablocks I. DOI: 10.1107/S1600536811003746/wn2420Isup2.hkl

Additional supplementary materials:  crystallographic information; 3D view; checkCIF report

## Figures and Tables

**Fig. 1.**
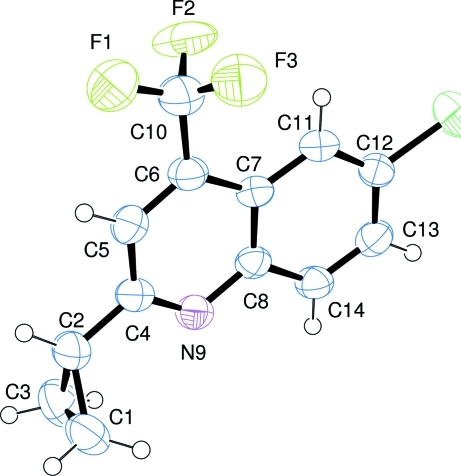
The molecular structure of the title compound. Displacement ellipsoids are drawn at the 50% probability level. Hydrogen atoms are shown as spheres of arbitrary radius.

**Fig. 2.**
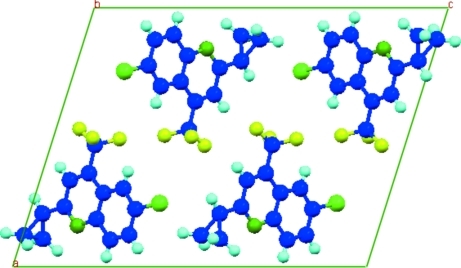
The packing of molecules, viewed down the b axis.

## supplementary crystallographic information

### Comment

1-Cyclopropyl-6-fluoro-1,4-dihydro-4-oxo-7-(1-piperazinyl)- 3-quinolinecarboxylic acid (ciprofloxacin) is a widely used broad-spectrum antibiotic, which is active against both Gram-positive  and Gram-negative bacteria (Kayser & Novak, 1987; Rudin *et al.*, 1984). 2,8-Bis(trifluoromethyl)quinolin-4-yl]-(2-piperidyl)methanol (mefloquin) is another popular quinoline derivative used in the treatment of malaria. Furthermore, studies have reported that it also possesses important structural features required for antimicrobial activity (Mao *et al.*, 2009; Bermudez *et al.*, 2004; Jayaprakash *et al.*, 2006). Quinoline is the essential structural feature found in these drugs and recently developed antimycobacterial drugs (Andries *et al.*, 2005). Thus, quinoline derivatives are good lead molecules to further develop drug candidates against mycobacterium tuberculosis and as antibacterial agents. On the basis of these observations, we have synthesized a quinoline derivative, with a cyclopropyl  group and a trifluoromethyl group as substituents, expecting that the newly designed hybrid molecule would exhibit some antibacterial activity. In this paper we report the crystal structure of 6-chloro-2-cyclopropyl-4-(trifluoromethyl)quinoline.

The asymmetric unit of the 6-chloro-2-cyclopropyl-4-(trifluoromethyl) quinoline contains one molecule (Fig. 1). The quinoline ring system makes a dihedral angle of 88.8 (2)° with the cyclopropyl ring. Bond distances and bond angles in the quinoline ring system are in good agreement with those observed in related crystal structures (Skörska *et al.*, 2005; Devarajegowda *et al.*, 2010; Li *et al.*, 2005). The packing of the molecules, when viewed along the *b* axis, is shown in Fig. 2.

### Experimental

A mixture of cyclopropyl acetylene (0.012 mol), anhydrous zinc(II) (0.012 mol), triethylamine (1.67 ml, 0.012 mol), and toluene (25 ml) was stirred at 50°C for 2 h and cooled to 25°C. 4-Chloro- 2-trifluoroacetylaniline (0.01 mol) was added and the reaction mixture was stirred at 25°C for 4 h, then at 50°C for 4 h. After cooling to room temperature, the mixture was added to water (10 ml) and extracted three times with ethyl acetate (20 ml). The combined organic phase was washed with brine and dried over anhydrous sodium sulfate. After removal of solvent, the residue was purified by column chromatography on silica gel (hexane/ethyl acetate; 20:1). M.p. 335 K.

### Refinement

All H atoms were placed at calculated positions; C—H = 0.93 Å for aromatic H, C—H = 0.97 Å for methylene H; C—H = 0.98 Å for methine H. They were refined using a riding model with *U*_iso_(H) = 1.2*U*_eq_(C).

### Crystal data

**Table:**

C_13_H_9_ClF_3_N	*F*(000) = 552
*M**_r_* = 271.66	*D*_x_ = 1.498 Mg m^−^^3^
Monoclinic, *P*2_1_/*c*	Melting point: 335 K
Hall symbol: -P 2ybc	Mo *K*α radiation, λ = 0.71073 Å
*a* = 13.8482 (19) Å	Cell parameters from 2105 reflections
*b* = 5.0534 (8) Å	θ = 2.4–25.0°
*c* = 18.048 (3) Å	µ = 0.33 mm^−^^1^
β = 107.503 (17)°	*T* = 293 K
*V* = 1204.5 (3) Å^3^	Plate, colourless
*Z* = 4	0.22 × 0.15 × 0.12 mm

### Data collection

**Table:**

Oxford Diffraction Xcalibur diffractometer	2105 independent reflections
Radiation source: Enhance (Mo) X-ray Source	946 reflections with *I* > 2σ(*I*)
graphite	*R*_int_ = 0.092
Detector resolution: 16.0839 pixels mm^-1^	θ_max_ = 25.0°, θ_min_ = 2.4°
ω scans	*h* = −16→16
Absorption correction: multi-scan (*CrysAlis PRO RED*; Oxford Diffraction, 2010)	*k* = −6→6
*T*_min_ = 0.942, *T*_max_ = 0.961	*l* = −21→21
11631 measured reflections	

### Refinement

**Table:**

Refinement on *F*^2^	Secondary atom site location: difference Fourier map
Least-squares matrix: full	Hydrogen site location: inferred from neighbouring sites
*R*[*F*^2^ > 2σ(*F*^2^)] = 0.041	H-atom parameters constrained
*wR*(*F*^2^) = 0.081	*w* = 1/[σ^2^(*F*_o_^2^) + (0.0334*P*)^2^] where *P* = (*F*_o_^2^ + 2*F*_c_^2^)/3
*S* = 0.78	(Δ/σ)_max_ = 0.004
2105 reflections	Δρ_max_ = 0.13 e Å^−^^3^
164 parameters	Δρ_min_ = −0.17 e Å^−^^3^
0 restraints	Extinction correction: *SHELXL97* (Sheldrick, 2008), Fc^*^=kFc[1+0.001xFc^2^λ^3^/sin(2θ)]^-1/4^
Primary atom site location: structure-invariant direct methods	Extinction coefficient: 0.0045 (8)

### Special details

**Table:**

Experimental. *CrysAlis PRO*, Oxford Diffraction Ltd., Version 1.171.33.55 (release 05–01–2010 CrysAlis171. NET) Empirical absorption correction using spherical harmonics, implemented in SCALE3 ABSPACK scaling algorithm.
Geometry. All s.u.'s (except the s.u. in the dihedral angle between two l.s. planes) are estimated using the full covariance matrix. The cell s.u.'s are taken into account individually in the estimation of s.u.'s in distances, angles and torsion angles; correlations between s.u.'s in cell parameters are only used when they are defined by crystal symmetry. An approximate (isotropic) treatment of cell s.u.'s is used for estimating s.u.'s involving l.s. planes.
Refinement. Refinement of *F*^2^ against ALL reflections. The weighted *R*-factor *wR* and goodness of fit *S* are based on *F*^2^, conventional *R*-factors *R* are based on *F*, with *F* set to zero for negative *F*^2^. The threshold expression of *F*^2^ > 2σ(*F*^2^) is used only for calculating *R*-factors(gt) *etc*. and is not relevant to the choice of reflections for refinement. *R*-factors based on *F*^2^ are statistically about twice as large as those based on *F*, and *R*- factors based on ALL data will be even larger.

### Fractional atomic coordinates and isotropic or equivalent isotropic displacement parameters (Å^2^)

**Table:**

	*x*	*y*	*z*	*U*_iso_*/*U*_eq_	
Cl1	0.24504 (7)	−0.53695 (16)	0.14138 (5)	0.0724 (3)	
F1	0.53230 (12)	0.2294 (4)	0.43406 (12)	0.1017 (8)	
F2	0.49335 (13)	0.1618 (4)	0.31203 (13)	0.0964 (7)	
F3	0.49773 (12)	−0.1595 (4)	0.38888 (11)	0.0896 (7)	
N9	0.15811 (17)	0.2481 (4)	0.35357 (14)	0.0463 (6)	
C1	0.1215 (2)	0.4767 (6)	0.49521 (17)	0.0629 (9)	
H1A	0.0846	0.3137	0.4779	0.076*	
H1B	0.1300	0.5228	0.5490	0.076*	
C2	0.2051 (2)	0.5449 (6)	0.46255 (18)	0.0612 (9)	
H2	0.2623	0.6398	0.4980	0.073*	
C3	0.1078 (2)	0.6908 (6)	0.43962 (19)	0.0695 (10)	
H3A	0.1077	0.8701	0.4588	0.083*	
H3B	0.0624	0.6610	0.3878	0.083*	
C4	0.2319 (2)	0.3623 (5)	0.40752 (17)	0.0486 (8)	
C5	0.3343 (2)	0.3113 (6)	0.41542 (17)	0.0542 (9)	
H5	0.3843	0.3978	0.4540	0.065*	
C6	0.3612 (2)	0.1390 (6)	0.36802 (17)	0.0463 (8)	
C7	0.2855 (2)	0.0029 (5)	0.30968 (16)	0.0401 (7)	
C8	0.1844 (2)	0.0691 (5)	0.30551 (16)	0.0405 (7)	
C10	0.4703 (3)	0.0937 (8)	0.3759 (2)	0.0678 (10)	
C11	0.3025 (2)	−0.1850 (5)	0.25749 (16)	0.0452 (8)	
H11	0.3683	−0.2290	0.2591	0.054*	
C12	0.2229 (2)	−0.3017 (5)	0.20490 (16)	0.0456 (8)	
C13	0.1232 (2)	−0.2394 (6)	0.19959 (16)	0.0489 (8)	
H13	0.0699	−0.3229	0.1630	0.059*	
C14	0.1044 (2)	−0.0553 (6)	0.24840 (16)	0.0464 (8)	
H14	0.0378	−0.0105	0.2442	0.056*	

### Atomic displacement parameters (Å^2^)

**Table:**

	*U*^11^	*U*^22^	*U*^33^	*U*^12^	*U*^13^	*U*^23^
Cl1	0.0857 (7)	0.0722 (6)	0.0640 (6)	−0.0003 (5)	0.0296 (5)	−0.0188 (5)
F1	0.0448 (12)	0.1346 (18)	0.1136 (18)	−0.0181 (12)	0.0057 (12)	−0.0505 (15)
F2	0.0542 (13)	0.148 (2)	0.1014 (19)	−0.0123 (12)	0.0445 (12)	0.0029 (14)
F3	0.0516 (12)	0.0939 (15)	0.1143 (18)	0.0143 (11)	0.0113 (11)	−0.0095 (13)
N9	0.0436 (15)	0.0528 (16)	0.0448 (16)	−0.0020 (14)	0.0165 (13)	−0.0026 (14)
C1	0.085 (3)	0.058 (2)	0.057 (2)	0.0067 (19)	0.0374 (19)	−0.0017 (19)
C2	0.049 (2)	0.071 (2)	0.066 (2)	−0.0092 (19)	0.0205 (19)	−0.024 (2)
C3	0.095 (3)	0.051 (2)	0.068 (3)	0.011 (2)	0.032 (2)	0.0001 (19)
C4	0.044 (2)	0.055 (2)	0.051 (2)	−0.0035 (17)	0.0208 (18)	−0.0058 (17)
C5	0.044 (2)	0.063 (2)	0.054 (2)	−0.0126 (17)	0.0135 (17)	−0.0157 (17)
C6	0.037 (2)	0.055 (2)	0.049 (2)	0.0002 (16)	0.0153 (17)	−0.0012 (16)
C7	0.036 (2)	0.0463 (19)	0.0404 (18)	−0.0017 (15)	0.0158 (15)	0.0033 (16)
C8	0.044 (2)	0.0452 (18)	0.0351 (18)	−0.0051 (16)	0.0169 (16)	0.0028 (15)
C10	0.051 (3)	0.078 (3)	0.077 (3)	−0.003 (2)	0.023 (2)	−0.013 (2)
C11	0.0381 (19)	0.0523 (19)	0.049 (2)	0.0010 (16)	0.0191 (17)	0.0017 (17)
C12	0.053 (2)	0.0478 (19)	0.0413 (19)	−0.0054 (17)	0.0221 (17)	−0.0045 (15)
C13	0.045 (2)	0.055 (2)	0.046 (2)	−0.0110 (17)	0.0128 (17)	−0.0012 (17)
C14	0.0364 (18)	0.056 (2)	0.048 (2)	−0.0007 (16)	0.0144 (17)	−0.0033 (17)

### Geometric parameters (Å, °)

**Table:**

Cl1—C12	1.741 (3)	C4—C5	1.406 (4)
F1—C10	1.329 (3)	C5—C6	1.349 (3)
F2—C10	1.330 (4)	C5—H5	0.9300
F3—C10	1.335 (3)	C6—C7	1.420 (3)
N9—C4	1.314 (3)	C6—C10	1.492 (4)
N9—C8	1.376 (3)	C7—C11	1.406 (3)
C1—C3	1.449 (4)	C7—C8	1.419 (3)
C1—C2	1.490 (4)	C8—C14	1.413 (3)
C1—H1A	0.9700	C11—C12	1.354 (3)
C1—H1B	0.9700	C11—H11	0.9300
C2—C3	1.481 (4)	C12—C13	1.392 (3)
C2—C4	1.482 (4)	C13—C14	1.359 (3)
C2—H2	0.9800	C13—H13	0.9300
C3—H3A	0.9700	C14—H14	0.9300
C3—H3B	0.9700		
			
C4—N9—C8	117.5 (3)	C5—C6—C10	120.2 (3)
C3—C1—C2	60.5 (2)	C7—C6—C10	119.8 (3)
C3—C1—H1A	117.7	C11—C7—C8	119.0 (3)
C2—C1—H1A	117.7	C11—C7—C6	126.1 (3)
C3—C1—H1B	117.7	C8—C7—C6	114.9 (3)
C2—C1—H1B	117.7	N9—C8—C14	117.0 (3)
H1A—C1—H1B	114.8	N9—C8—C7	124.4 (3)
C3—C2—C4	120.8 (3)	C14—C8—C7	118.6 (3)
C3—C2—C1	58.35 (19)	F1—C10—F2	106.5 (3)
C4—C2—C1	120.1 (3)	F1—C10—F3	105.9 (3)
C3—C2—H2	115.3	F2—C10—F3	105.7 (3)
C4—C2—H2	115.3	F1—C10—C6	113.0 (3)
C1—C2—H2	115.3	F2—C10—C6	112.2 (3)
C1—C3—C2	61.12 (19)	F3—C10—C6	113.0 (3)
C1—C3—H3A	117.7	C12—C11—C7	119.9 (3)
C2—C3—H3A	117.7	C12—C11—H11	120.0
C1—C3—H3B	117.7	C7—C11—H11	120.0
C2—C3—H3B	117.7	C11—C12—C13	122.1 (3)
H3A—C3—H3B	114.8	C11—C12—Cl1	119.5 (2)
N9—C4—C5	122.0 (3)	C13—C12—Cl1	118.5 (2)
N9—C4—C2	118.4 (3)	C14—C13—C12	119.3 (3)
C5—C4—C2	119.6 (3)	C14—C13—H13	120.3
C6—C5—C4	121.1 (3)	C12—C13—H13	120.3
C6—C5—H5	119.4	C13—C14—C8	121.1 (3)
C4—C5—H5	119.4	C13—C14—H14	119.5
C5—C6—C7	120.0 (3)	C8—C14—H14	119.5
			
C3—C1—C2—C4	−109.7 (3)	C6—C7—C8—N9	−0.6 (4)
C4—C2—C3—C1	108.6 (3)	C11—C7—C8—C14	−0.5 (4)
C8—N9—C4—C5	1.6 (4)	C6—C7—C8—C14	179.3 (2)
C8—N9—C4—C2	−176.8 (2)	C5—C6—C10—F1	2.5 (5)
C3—C2—C4—N9	−27.5 (4)	C7—C6—C10—F1	−178.5 (3)
C1—C2—C4—N9	41.4 (4)	C5—C6—C10—F2	−118.0 (3)
C3—C2—C4—C5	154.1 (3)	C7—C6—C10—F2	61.0 (4)
C1—C2—C4—C5	−137.1 (3)	C5—C6—C10—F3	122.6 (3)
N9—C4—C5—C6	−0.8 (5)	C7—C6—C10—F3	−58.3 (4)
C2—C4—C5—C6	177.6 (3)	C8—C7—C11—C12	−0.7 (4)
C4—C5—C6—C7	−0.9 (4)	C6—C7—C11—C12	179.5 (3)
C4—C5—C6—C10	178.2 (3)	C7—C11—C12—C13	0.9 (4)
C5—C6—C7—C11	−178.7 (3)	C7—C11—C12—Cl1	−179.23 (19)
C10—C6—C7—C11	2.2 (4)	C11—C12—C13—C14	0.2 (4)
C5—C6—C7—C8	1.5 (4)	Cl1—C12—C13—C14	−179.7 (2)
C10—C6—C7—C8	−177.6 (3)	C12—C13—C14—C8	−1.5 (4)
C4—N9—C8—C14	179.1 (2)	N9—C8—C14—C13	−178.4 (2)
C4—N9—C8—C7	−0.9 (4)	C7—C8—C14—C13	1.6 (4)
C11—C7—C8—N9	179.5 (2)		
